# Antagonistic Microbial Interactions: Contributions and Potential Applications for Controlling Pathogens in the Aquatic Systems

**DOI:** 10.3389/fmicb.2017.02192

**Published:** 2017-11-14

**Authors:** Judith Feichtmayer, Li Deng, Christian Griebler

**Affiliations:** ^1^Institute of Groundwater Ecology, Helmholtz Zentrum München GmbH, Neuherberg, Germany; ^2^Institute of Virology, Helmholtz Zentrum München GmbH, Neuherberg, Germany

**Keywords:** pathogens, antimicrobial substances, grazing, bacteriophages, BALO, antagonistic interactions, aquatic environment

## Abstract

Despite the active and intense treatment of wastewater, pathogenic microorganisms and viruses are frequently introduced into the aquatic environment. For most human pathogens, however, this is a rather hostile place, where starvation, continuous inactivation, and decay generally occur, rather than successful reproduction. Nevertheless, a great diversity of the pathogenic microorganisms can be detected, in particular, in the surface waters receiving wastewater. Pathogen survival depends majorly on abiotic factors such as irradiation, changes in water ionic strength, temperature, and redox state. In addition, inactivation is enhanced by the biotic interactions in the environment. Although knowledge of the antagonistic biotic interactions has been available since a long time, certain underlying processes and mechanisms still remain unclear. Others are well-appreciated and increasingly are applied to the present research. Our review compiles and discusses the presently known biotic interactions between autochthonous microbes and pathogens introduced into the aquatic environment, including protozoan grazing, virus-induced bacterial cell lysis, antimicrobial substances, and predatory bacteria. An overview is provided on the present knowledge, as well as on the obvious research gaps. Individual processes that appear promising for future applications in the aquatic environment are presented and discussed.

## Introduction

Pathogenic microorganisms are frequent visitors, or even inhabitants, of the aquatic environments. Their paths of entry into the natural water cycle are manifold; however, the primary sources include treated and untreated wastewater, as well as manure applied to the agricultural lands. Wastewater from households and hospitals undergoes a moderate reduction of pathogens when it is collected and treated in the sewage treatment plants, approximately 1 to 3 orders of magnitude; therefore, we know that higher numbers of pathogens are continuously released into the recipient surface waters, in particular in times of increased bacterial and viral infections in the human population (George et al., 2002; Reynolds and Barrett, 2003; Gerba and Smith, 2005; Arnone and Walling, 2007). From these recipient water bodies, pathogens are then distributed into the connected surface waters, such as rivers and lakes, as well as groundwater. In rural areas and less developed countries, pathogens enter the terrestrial and aquatic environments through active discharge or accidental loss (e.g., leakages from onsite sanitation systems). Direct entry into groundwater and surface water also occurs when the manure disposed on agricultural lands encounters heavy precipitation, and surface run-off and seepage occur through the unsaturated zone.

Extreme hydrological events, such as floods, may become more frequent in the future due to ongoing global change, increasing the pressure on the already stressed terrestrial and aquatic environments. Insufficiently treated manure, wastewater, and discharge from the sewage treatment plants are frequently spread to the water sources used for drinking water production or recreation (Schwarzenbach et al., 2010). Once present in the aquatic environment, pathogens become a frequent cause of outbreaks of water-borne diseases, constituting a severe risk for human health (Mounts et al., 2000; Albinana-Gimenez et al., 2006; Jiang, 2006).

For most human pathogens, the aquatic environment is a hostile place, where they starve, are continuously inactivated, and eventually decay, rather than reproduce successfully. Despite these hostile conditions, a great diversity of pathogenic microorganisms are often detected, in particular, in the surface waters receiving wastewater (Seidel et al., 2016), and occasionally persist for several years (Krauss and Griebler, 2011).

Prominent examples of pathogenic bacteria regularly found in the surface and subsurface waters include *Escherichia coli, Vibrio cholerae, Yersinia enterocolitica*, as well as species of the genera *Salmonella* and *Legionella* (Macler and Merkle, 2000; Krauss and Griebler, 2011; Seidel et al., 2016). Some pathogenic bacteria, such as *Pseudomonas aeruginosa, E. coli*, as well as species of the genera *Legionella* and *Mycobacterium*, have been found repeatedly surviving, and even multiplying, outside their human hosts (Vital et al., 2007, 2008); however, in most cases, in order to propagate, several human pathogenic bacteria require specific conditions (favorable temperatures, available nutrients, specific redox states) that are rarely fulfilled simultaneously in the environment (Riffard et al., 2001; Leclerc et al., 2002; Brookes et al., 2004; Vital et al., 2008).

In the aquatic environment, diversity among the water-borne pathogens is highest with enteric viruses (Wyn-Jones and Sellwood, 2001). Being obligate intracellular parasites, viruses depend on their specific hosts for propagation; therefore, human pathogenic viruses do not have a natural host in the environment, and thus, are only able to persist to some extent, but not to replicate. Upon an acute infection, the enteric viruses like *Coxsackievirus, Norovirus*, Hepatitis A, and Hepatitis E, or respiratory viruses like *Adenovirus* or *Echovirus*, are released in higher numbers via feces into the wastewater, where they eventually end up in the environment (Macler and Merkle, 2000; Fong and Lipp, 2005). When encountering a new host, generally only few, sometimes only one, intact particle is needed to provoke an infection (Zwart et al., 2009). Moreover, viral particles may maintain their infectivity over long durations, even longer than enteric bacteria under certain circumstances (Fong and Lipp, 2005; Krauss and Griebler, 2011; Stevenson et al., 2015). For example, *E. coli* needed 250 days to become undetectable by plate counts, whereas for *Poliovirus*, persistence times of 550 days in groundwater have been reported (Althaus et al., 1982; Filip et al., 1986).

The fate of pathogens in the aquatic environment is majorly determined by a broad range of abiotic factors, and indeed, these factors are the core drivers of pathogenic inactivation and degradation (e.g., Burkhardt et al., 2000; Sinton et al., 2002; Brookes et al., 2004). In the surface waters, UV irradiation is a major factor responsible for the effective inactivation and decay of microorganisms, although it may occur at different rates (Jacquet and Bratbak, 2003; Hijnen et al., 2006). With respect to soils and sediments, adsorption to the sediment matrix causes attenuation that can be reversible or irreversible (Jin et al., 2000; Chu et al., 2001; Blanford et al., 2005; Brusseau et al., 2005). Moreover, the hydrophobicity of soils and sediments, as well as their porosity, grain size distribution, and pore water chemistry (such as pH or ionic strength), are additional factors that influence the bacterial and viral retention (Gordon and Toze, 2003; Klitzke et al., 2005; Cao et al., 2010; Sadeghi et al., 2011).

**FIGURE 1:**
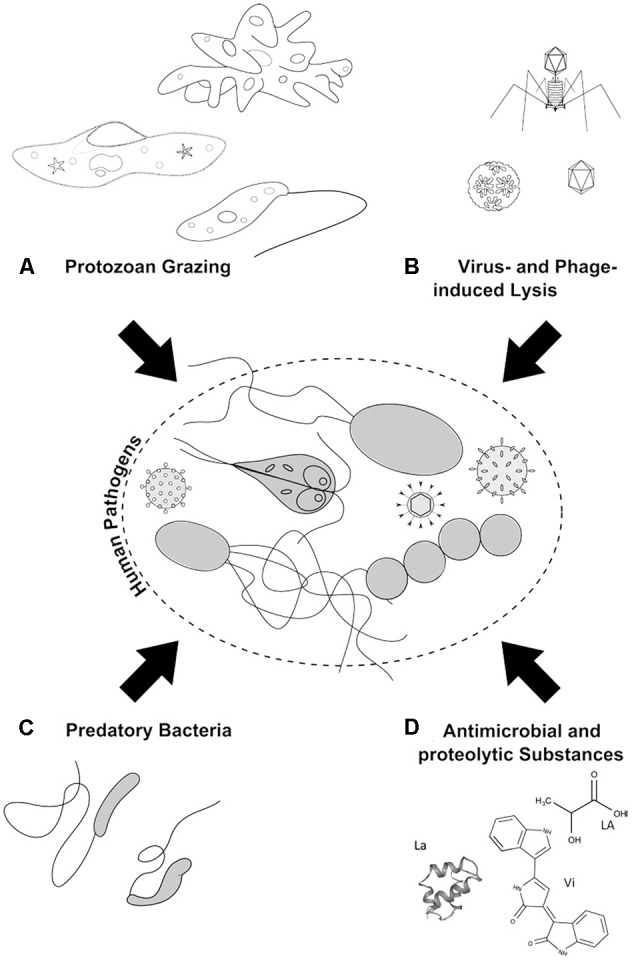
Antagonistic microbial interactions: **(A)** Protozoan grazing on pathogenic microorganisms and viruses by amoeba, ciliates, and flagellates. **(B)** Phage-induced lysis of pathogenic bacteria and protozoa. **(C)** Predation of pathogenic bacteria by BALOs. **(D)** Microbial chemical war-substances with antimicrobial activity like lactic acid (LA) and violacein (Vi), as well as proteolytic substances, such as lacticin (La), are produced and excreted by bacteria to inhibit and kill/lyse opponents.

Little consideration has been given to the influence of natural microbial antagonists, such as protozoa, bacteria, and phages, on the fate of incoming pathogens in the aquatic environment. Microbial communities in the environment form a complex interactive network of commensalism, antagonism, and parasitism (Hibbing et al., 2010), thus, biotic interactions are essential determinants of the natural microbial communities (Vos et al., 2009). Relationships between species (e.g., bacteria–bacteria) and between members of different trophic levels (guilds) within a food web (e.g., phage–bacteria, protozoa–bacteria, protozoa–phage) may be mutualistic or antagonistic, both fostering community development through co-evolutionary processes. Autochthonous microorganisms not only have an advantage over introduced pathogens in terms of competitiveness but also are assumed to contribute actively to the pathogen inactivation and elimination. With regard to this, initial evidences were collected in the early 20th century revealing that persistence times of pathogenic microorganisms are significantly shorter in the biologically active soil compared to the sterile soil (Gärtner, 1915). Since then, numerous observations from laboratory experiments and a few field studies have supported the assumption that microbially active soils reduce the amount of introduced pathogenic microorganisms; however, these studies have a mostly descriptive character, and the specificity as well as the extent of this biotic inactivation is not well-understood (Cutler, 1923; Postma et al., 1990; van Veen et al., 1997). This is, in particular, true regarding the combined action of several antagonistic processes that have received little attention to date. To fill this gap, our review aims to provide an overview of the biotic interactions between the autochthonous microbes in the aquatic environment and the pathogens that are being introduced. Individual antagonistic interactions (i.e., biotic mechanisms acting negatively on pathogens) are emphasized here (**Figure 1**) including: (i) protozoan grazing on prokaryotes and viruses, (ii) the virus- and phage-induced lysis of bacteria (prokaryotes) and protozoa, (iii) the bacterial production and release of antimicrobial (e.g., bacterial toxins) and proteolytic substances, and (iv) the activity of predatory bacteria (e.g., *Bdellovibrio*). A discussion follows regarding the possible role(s) of these antagonistic processes in the fate of pathogens in aquatic systems. Eventually, the present options and limitations are discussed regarding human use of antagonistic microbial processes to reduce the number of human pathogens in the aquatic environment.

## Antagonistic Interactions and Applications

### Protozoan Grazing on Pathogenic Bacteria and Viruses

In the natural aquatic ecosystems, mortality of prokaryotes is caused, to a great extent, by protozoan grazing (Menon et al., 2003). Ingestion rates vary widely across the different groups of protozoa (amoebae, heterotrophic nanoflagellates, and ciliates), depending on their feeding behavior, prey size, and prey abundance (Pernthaler, 2005). The daily reduction of the bacterial standing stock by ciliate grazing may range between 1 and 8% (Kemp, 1988; Wieltschnig et al., 2003; Tuorto and Taghon, 2014). Clearance rates of heterotrophic nanoflagellates have been estimated to account for up to 50%, although grazing efficiencies vary strongly according to the study (Weisse and Müller, 1990; Wieltschnig et al., 2003; Bettarel et al., 2004). While ciliates and heterotrophic nanoflagellates are effective grazers in the open water column, amoeba graze primarily on biofilms (Zhang et al., 2014). Bacterial and viral losses through grazing are most often influenced by the trophic status of the water source and by season (Jacquet et al., 2005). Losses of the bacterial standing stock in the eutrophic lakes have been measured at values up to 28%, whereas researchers have reported losses in the oligotrophic lakes of up to 70% (Šimek et al., 1997; Domaizon et al., 2003).

**FIGURE 2:**
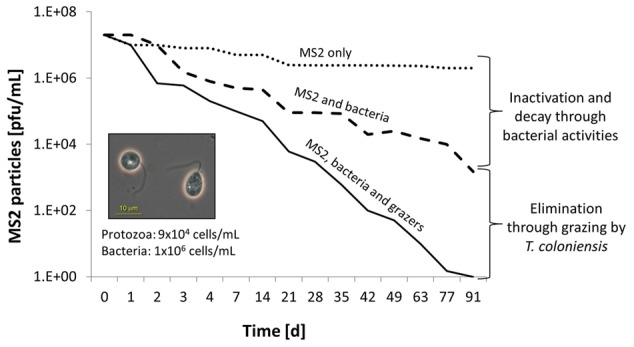
Microbially active water leads to the reduction of allochthonous viruses. The model coliphage MS2 is reduced in the presence of native groundwater bacteria (dashed line) and the benthivorous heterotrophic nanoflagellate, *Thaumatomonas coloniensis* (solid line). The dotted line reveals the virus-only control (modified from Deng et al., 2014).

Although other small protozoa and bacteria are the favored food sources, studies have reported that *Tetrahymena* and other ciliates, heterotrophic nanoflagellates, and amoeba take up non-attached viral particles as well (Groupé and Pugh, 1952; Knorr, 1957, 1960; Suttle and Chen, 1992; Gonzalez et al., 1993; Bettarel et al., 2005; Evans and Wilson, 2008; Bouvy et al., 2011). Deng et al. (2014) compared the reduction in the population of the model bacteriophage MS2 in the presence of three heterotrophic flagellates: the filter-feeding flagellate *Salpingoeca* sp.; the benthivorous grazer *Thaumatomonas coloniensis*; and the active raptorial feeder *Goniomonas truncate*. The experiment was performed in the presence of a natural bacterial community in groundwater. Grazing by *Salpingoeca* sp. or *T. coloniensis*, decreased the MS2 titer by six orders of magnitude within a 90-day period (**Figure 2**). In the absence of protozoa, a reduction of only 2 log units was observed, and that was attributed to the antagonistic activities of the bacterial community. Although ingested, viruses only marginally contribute to the protozoan diet in terms of carbon (Deng et al., 2014). Hennemuth et al. (2008) demonstrated that protozoa may directly reutilize viral amino acids for their own protein biosynthesis.

As grazing efficiency is strongly affected by prey size, motility, nutritional quality, and cell-surface characteristics, there is debate regarding whether the pathogenic bacteria and viruses are consumed by protozoa selectively, or merely by chance. Early evidence was provided by Bahr (1954) that there is some selective discrimination. A mixture of different bacterial strains was offered to various ciliate cultures. After some days, the individual ciliate cells were picked and transferred into a saline solution to induce cell lysis. Bacteria released from the bursting cells were cultured on selective agar (endoagar). Although *E. coli* could be isolated in the early stages of the experiment, at later time points only *Bacillus subtilis*, and to a lesser extent *Staphylococcus aureus*, were found present inside the ciliates. Moreover, besides the evidence for selective avoidance, *E. coli* was even found harmful for the ciliate grazers, as uptake of these species led to protozoan decay. It is important to consider that not all particles ingested by the protozoa are inactivated and/or digested. Occasionally, some bacteria, like *V. cholerae* or *Legionella pneumophila*, have revealed resistance to phagocytosis (e.g., by preventing the fusion of the lysosome with the phagosome, thus avoiding digestion) and may even replicate within amoebas or cause the death of their grazers (Barker and Brown, 1994; Kirby et al., 1998; Hägele et al., 2000; Greub and Raoult, 2004; Abd et al., 2007). Co-evolutionary selective forces continuously drive the development of bacterial-evading mechanisms by altering the cell surface molecules, cell morphology, speed of motility, biofilm formation, or toxin release (Matz and Kjelleberg, 2005; Matz et al., 2005; Pernthaler, 2005; Siddiqui and Khan, 2012). Similar anti-grazing mechanisms are not known for viruses, to date, but may indeed exist.

Protozoa play a key role in balancing the bacterial populations not only in natural aquatic systems but also in the wastewater treatment plants or other kinds of bioreactors. In particular, during the biological phase of the wastewater treatment process, protozoa are important for the flocculation and reduction of bacterial biomass (Lee and Welander, 1996; Pauli et al., 2001). The effectiveness of protozoa as biocontrol agents against human pathogenic bacteria and viruses in both manmade and natural aquatic systems depends upon many factors, including protozoan abundance, growth and grazing rates, predation (in) specificity, pathogen abundances and growth rates, as well as rates of predation on protozoa by higher organisms (e.g., copepods) (Brabrand et al., 1983; Sigee et al., 1999). Improved removal of enteric bacteria due to protozoan grazing has been observed in the biological filters (Stevik, 1998). Although concentrations of undesired bacteria and/or viruses, as well as of grazers, are less in the natural aquatic environments, similar effects may be expected; however, no conclusive data are available at this point.

### Viruses and Phage-Induced Lysis of Pathogens

Viruses that prey exclusively on prokaryotes are called bacteriophages or phages. In the aquatic environment, phages outnumber bacteria and archaea by 10-fold or more (Fuhrman, 1999) and phage-induced lysis of prokaryotes accounts for 5–50% of the day-to-day bacterial mortality (Fuhrman and Noble, 1995; Wommack and Colwell, 2000; Weinbauer, 2004; Suttle, 2007; Brussaard et al., 2010). Phages generally display certain specificity for a host; however, that range can be very narrow, for a particular species only, or relatively broad, including various species within a common grouping. This includes human pathogenic bacteria. It is assumed that every organism has its own subset of viruses to which it is susceptible. In a study undertaken by Khan et al. (2002), it was reported that viral predation can even encompass both Gram-negative and Gram-positive bacteria. Indeed, lytic phages influence the microbial diversity and population structures, thus adding a significant selective pressure on the microbial communities (Letarov and Kulikov, 2009; De Paepe et al., 2014). In the oligotrophic environments, phage-induced lysis may stabilize the co-existence of bacteria by avoiding the overgrowth of a single species (a scenario known as the “killing-the-winner” theory) (Thingstad and Lignell, 1997; Shapiro et al., 2010; Winter et al., 2010). Additionally, fitness costs for carrying phage-resistance genes in nutrient-poor environments, such as groundwater, are comparably high. This indicates that, in this type of environment, phages cause minor, but continuous long-term diminishing effects on the bacterial biomass (Lopez-Pascua and Buckling, 2008). Likewise, the evolution of phage resistance in these environments reveals a strong association with the presence of co-occurring phages (Gómez and Buckling, 2011). As a type of protective function, certain bacteria may organize themselves into biofilms, which are more difficult for phages to access; however, as a remedy to the bacterial solution, some phages have evolved polysaccharide depolymerases attached to their tail fibers that digest the bacterial extracellular polymeric substances (EPSs), gaining access to bacterial cell surfaces (Adams and Park, 1956; Hughes et al., 1998a,b). Temperate phages (or prophages) can contribute to phenotypic changes via horizontal gene transfer, driving bacterial evolution and adaptation to new habitats, and this is often accompanied by an increase in bacterial virulence (Jiang and Paul, 1998). Both human-pathogenic serotypes of *V. cholerae* (O1 and O139) can acquire two pivotal virulence factors (toxin-co-regulated pilus and cholera toxin) that were found being mediated by phages (Waldor and Mekalanos, 1996; Karaolis et al., 1999). Indeed, most toxin-coding genes are linked to a lysogenic lifestyle, as with the diphtheria toxin or the cholera toxin, leading to a great risk of emerging new pathogenic bacteria (Freeman, 1951; Waldor and Mekalanos, 1996; Brüssow et al., 2004; Tinsley et al., 2006).

Of all the participants acting in antagonistic microbial interactions, viruses (and here mainly bacteriophages) probably represent the most powerful ones. Isolated bacteriophages were used in the early 1920s to treat the pathogenic bacteria in humans, a therapy that has recently regained attention due to the growing number of multidrug-resistant pathogenic bacteria (Thiel, 2004; Viertel et al., 2014). To date, lytic activities of phages against a broad variety of pathogenic bacteria have been reported (e.g., against *S. aureus, P. aeruginosa, Salmonella enterica, V. cholerae*, or *E. coli*) (Slopek et al., 1987; Capparelli et al., 2010; Ceyssens and Lavigne, 2010).

**FIGURE 3:**
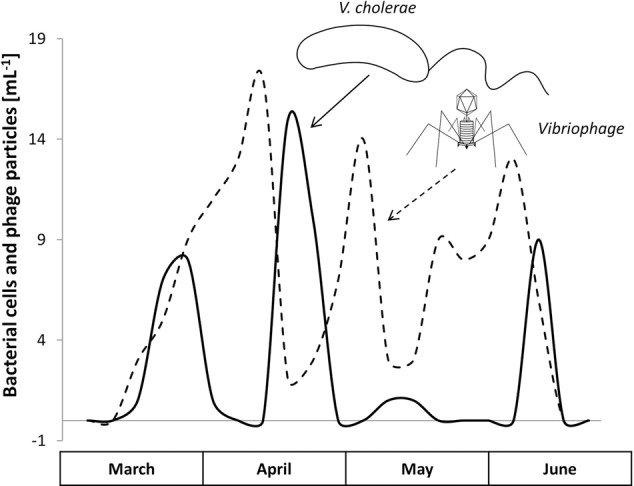
The natural occurrence and the Lotka–Volterra dynamics of *Vibrio cholerae* O1 (solid line) and its vibriophage (dashed line) during the predominant months, March to June, Hooghly River, West Bengal (modified from Mookerjee et al., 2014).

A 1-year surveillance study by Mookerjee et al. (2014) impressively presented data of a phage controlling a human pathogen that was indigenous to an aquatic environment. The team monitored the dynamics of the toxigenic *V. cholerae* strain O1 and its lytic phage (vibriophage) at two different sites of the Hooghly River in West Bengal over the three seasons, summer, monsoon, and winter. With an increasing abundance of *V. cholerae*, the corresponding phage titer increased, then leading to a responsive decline in the *V. cholerae* load (**Figure 3**). These repeating patterns strikingly underline the potential control that phages may render on pathogenic bacteria in a natural setting.

Active “phage therapy” in the environment has been suggested for several years. At present, phages are used routinely for the biocontrol of herbal or food borne pathogens, or for decontamination in aquacultures and food industries (Nakai and Park, 2002; Balogh et al., 2010; Goodridge and Bisha, 2011). The rapid generation time of cyanophages, for example, makes them attractive agents for controlling the toxic and bloom-forming cyanobacteria (Sigee et al., 1999 and references therein). When isolated from lake water and treated with a natural viral cocktail, *Microcystis aeruginosa* decreased in abundance by 95% within only a few days (Tucker and Pollard, 2005). In the aforementioned study, two phages displaying a T7-like morphology and belonging to the *Podoviridiae* group (short tails) were assumed to be responsible for killing the cyanobacterial strain. Yoshida et al. (2006) isolated a cyanophage (Ma-LMM01) that specifically infected and killed *Microcystis aeruginosa*. Baudoux and Brussaard (2005) isolated several lytic viruses from freshwater infecting the eukaryotic algae *Phaeocystis globosa*, an abundant and harmful, bloom-forming phytoplankton. Phages infecting *Vibrio coralliilyticus* and *Thalosomonas loyaeana*, both aggressive coral pathogens, were isolated and applied to curing infected corals (Efrony et al., 2007; Cohen et al., 2013). Another example of beneficial phage use is the dewatering process of sludge in wastewater treatment plants, which is an important process for condensing the sludge volume. High levels of EPS (up to 99% water content) are problematic in this step of the treatment, generally interfering with effective volume reduction (Costerton, 1999). The presence of some extensive EPS producers, like *Zoogloea* and *Thauera*, may be controlled by the application of selective bacteriophages (Kang et al., 1989; Thomas et al., 1993; Sanin and Vesilind, 1994). Another interesting feature for phage application has been observed recently by Chan et al. (2016). This group isolated a naturally occurring phage that forces a desired genetic trade-off between phage and antibiotic resistance, thus favoring a development toward increased antibiotic sensitivity for *P. aeruginosa* in the presence of this particular phage. Besides the successful application of phages in patients and other hot spots of pathogens in the laboratory and the environment, the effectiveness of bacteriophages selectively inactivating and killing target hosts in natural settings remains unclear and co-evolutionary mechanisms of hosts’ phage resistance needs further attention as it may limit a long-term application (see below).

### Bacterial Release of Toxins and Proteolytic Substances

In complex and diverse communities, competition for nutrients and space is high. Interspecific competition between prokaryotes often is mediated by the use of a variety of antimicrobials, such as secondary metabolites (e.g., lactic acids from lactobacilli), extracellular enzymes (e.g., lysozymes, exotoxins, bacteriocins), or antibiotics (e.g., streptomycin, tetracycline, or vancomycin) (Jack et al., 1995; De Boer et al., 2005; Riley and Chavan, 2007; Hibbing et al., 2010; de Lima Procópio et al., 2012). While we all got accustomed to the use of antimicrobial substances, such as antibiotics, against pathogens, we often forget that these substances are naturally produced by microbes, giving them a competitive advantage. Indeed, natural microbial communities have a yet unrecognized arsenal of substances that they apply daily in their “microbial war” (Hibbing et al., 2010). Bacteriocin production, for example, is found in a vast majority of bacteria (e.g., within the genera *Myxococcus, Lysobacter*, and *Bacillus*). Bacteriocins, such as colicin, are primarily active against closely related species, and work by degrading the antagonist’s inner membrane or nucleic acids. Gram-negative bacteria, in particular, lack a specific secreting system for bacteriocins; therefore, the release of these substances occurs via their own cell lysis. This indicates that only a small fraction of the bacterial population produces bacteriocins, thus providing a competitive edge to their population (Cascales et al., 2007). Nevertheless, for Gram-positive bacteria, bacteriocin production is not necessarily lethal, as some express a bacteriocin-specific transport system that is used for shuffling the antimicrobial back out of the cell (Riley and Wertz, 2002). The expression of antimicrobial substances generally occurs in the stationary phase of the bacterial growth cycle, when they are running short of nutrients. These compounds may enable or disable the invasion of a strain into an established community; they may provide the release of nutrients by cell lysis, and they may even affect the interbacterial communication (e.g., quorum sensing) (Miller and Bassler, 2001; Riley and Wertz, 2002).

**FIGURE 4:**
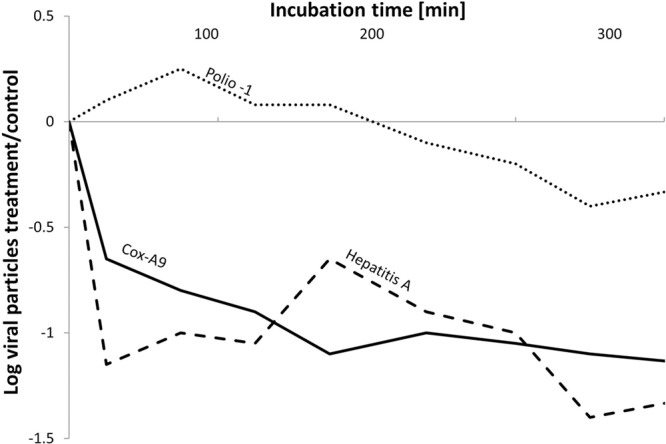
Effects of the extracellular activity from *Pseudomonas aeruginosa* on the persistence of the pathogenic viruses, Hepatitis A (dashed line), Cox-A9 (solid line), and Polio-1 virus (dotted line), during coincubations for 400 min. The Y-axis represents the calculated log values of virus titer in the *P. aeruginosa* incubations versus the virus concentration in a bacteria-free control (modified from Nasser et al., 2002).

Early studies on “lytic” bacteria emphasized their potential in controlling specific groups of microbes; however, those studies were primarily descriptive. Hirsch and Rades-Rohkohl (1983) isolated aerobic bacteria from groundwater and monitored their ability to reduce *E. coli* K12 numbers using a simple agar overlay method. From the 214 different bacterial isolates tested, 24% revealed inhibitory/lytic effects against *E. coli*. In total, 39% of the isolates displayed negative interactions against the fecal pathogen. As another example of potentially controlling cyanobacteria in aquatic environments, scientists found a direct statistical correlation between the chlorophyll-a concentration, the cyanobacterial biomass, and the abundance of lytic bacteria (Daft et al., 1975; Fallon and Brock, 1979). Similarly, the filtrate of different actinomycetes (e.g., *Streptomyces* sp.) revealed antimicrobial properties against 50% of more than 400 prokaryotic strains tested (Safferman and Morris, 1962). Similar studies on antimicrobial substances released by algae and actinomycetes reported not only antimicrobial effects but also antiviral effects (e.g., *Coxsackievirus* or *Poliovirus*) (Husmann, 1966; Cliver and Herrmann, 1972; Daubner, 1972; Herrmann and Cliver, 1973; Rehse, 1977). Nasser et al. (2002) examined the potential for proteases and elastases produced by Pseudomonads to reduce different viral titers, and found that the effects were dependent on the virus type (**Figure 4**). *Cox-A9 virus* and *Hepatitis A virus* were significantly affected by the presence of extracellular bacterial enzymes, whereas *Polio-1 virus* remained unaffected. Although similar in size, the viruses were characterized by pronounced differences in the compositions of their capsid proteins, as evidenced by differences in the isoelectric points. Accordingly, different viruses may react in a distinct manner in the presence of extracellular enzymes (Nasser et al., 1991, 2002).

Some reports are available on the bacteriocin responses against certain medically important human pathogenic Gram-negative bacteria (e.g., *Campylobacter, Heliobacter*, and *Neisseria*) (Mota-Meira et al., 2000). In the food industry, antimicrobials are used routinely as preservative agents and for the reduction of specific unwanted germs (Burnham et al., 1981; Riley and Gordon, 1992; Lin and McBride, 1996; Gautam and Sharma, 2009). For example, Nisin is a bacteriocin produced by *Lactococcus lactis* spp., and is used worldwide against a wide variety of Gram-positive bacteria (e.g., lactic acid bacteria) or heat-resistant bacterial spores (e.g., *Clostridium botulinum*) (Brewer et al., 2002; López-Pedemonte et al., 2003; Sobrino-López and Martín-Belloso, 2006; Lucera et al., 2012). Lysozymes are another type of antimicrobial compounds used specifically against Gram-positive bacteria, because they act by hydrolyzing the murein layer (Cunningham et al., 1991). The examples we have described here only provide insight into the actions of single antimicrobial substances that have been tested in the laboratory settings or applied under controlled conditions. Environmental-based studies, including those for human bacterial pathogens, are scarce. The effectiveness of intrinsically produced antimicrobial compounds at ambient concentrations in a heterogeneous and complex aquatic environment, as well as the co-evolving development of resistance, remains unclear and a better understanding is urgently needed (see below).

### Activity of Predatory Bacteria (Bdellovibrio-Type Feeding)

An antagonistic interaction that is rarely considered is the predation of bacteria by other bacteria. Bacteria that share this feeding mode are commonly described as “*Bdellovibrio* and like organisms” (BALO), with *Bdellovibrio* being the most studied and best characterized organism in this group. BALOs can be found in several environments including soils, waters of various qualities, and in wastewater treatment plants (Martin, 2002). *Bdellovibrio* is a Gram-negative Deltaproteobacterium, known for invading the periplasm of other bacteria. Suitable prey comprise mostly Gram-negative, planktonic, or attached bacteria (Dashiff et al., 2010). *Bdellovibrio* attacks by entering the periplasm of its prey, where it forms a bdelloplast, septates, and finally lyses its prey, releasing progeny cells (Stolp and Starr, 1963; Rendulic et al., 2004; Lambert et al., 2006; Davidov and Jurkevitch, 2009; Sockett, 2009). The bdelloplast serves as a protective shield against phototoxic and chemical damage or phage attack (Friedberg, 1977; Markelova, 2002). Other, less extensively studied BALOs, like *Micavibrio, Ensifer, Vampirococcus*, or *Daptobacter* have evolved different feeding behaviors (Guerrero et al., 1986; Yair et al., 2003; Davidov et al., 2006; Dashiff et al., 2010). *Micavibrio*, for example, is an Alphaproteobacterium that attaches to the surface of various planktonic and sessile bacteria (*Burkholderia, Enterobacter, Klebsiella, Pseudomonas*, etc.) without entering the prey, but instead, works to exhaust it from the outside (Davidov et al., 2006; Dashiff et al., 2010; Koval et al., 2013). While the exact mechanisms are not yet clear, a transcriptome analysis has provided the first evidence for the involvement of porins that may facilitate the uptake of metabolites derived from the degrading prey cells (Wang et al., 2011). Another extracellular predation strategy is applied by *Vampirococcus*, where the predator attaches via cytoplasmic bridge structures to the cell membrane of *Chromatium*, a phototrophic purple sulfuric bacterium living in freshwater. Subsequently, the introduction of hydrolytic enzymes leads to the degradation of the prey’s cytoplasm and the ingestion of its contents (Guerrero et al., 1986; Martin, 2002). Another example is *Daptobacter*, a Gram-negative, facultative anaerobic freshwater bacterium, that is also endobiotic, meaning it resides and replicates within the cytoplasm of its prey (e.g., the phototrophic *Chromaticeae*) (Guerrero et al., 1986).

Given the aforementioned highlighted details, our understanding of bacterivorous bacteria is still far from complete. There is, for example, still no proof for how BALOs are attracted to suitable prey. Chemotaxis toward certain amino acids and attraction to high bacterial concentrations, prey or not, seem to play important roles (LaMarre et al., 1977; Straley and Conti, 1977; Rendulic et al., 2004); however, it is not clear at this point (i) how they identify and distinguish their Gram-negative prey from Gram-positive bacteria or particles, (ii) how they come into contact with their prey, or (iii) how they manage to survive changes in osmolarity or pH that would be prevalent when encountering the periplasm of their victims (Rendulic et al., 2004; Sockett, 2009). In particular, BALOs’ preferred temperature range is 18–30°C, which questions its activity in cold aquatic habitats, such as the deep sea or groundwater in temperate regions (Filip et al., 1991; Dashiff et al., 2010).

The ability for bacterivorous bacteria to significantly reduce the pathogenic bacteria *in vitro* and *in vivo* has raised high expectations. Dashiff et al. (2010) confirmed the activity of *Bdellovibrio bacteriovorus* and *M. aeruginosavorus* strains against several pathogenic bacterial genera, like *Aeromonas, Burkholderia, Enterobacter, Salmonella, Shigella, Vibrio*, and *Yersinia.* Moreover, a strong reduction potential for the predatory bacteria against multidrug-resistant *Acinetobacter baumannii, E. coli, Klebsiella pneumoniae, P. putida*, and *P. aeruginosa* was indicated by Kadouri et al. (2013). Two experimental treatments testing the application of bacterivorous bacteria have proven successful: (1) oral applications of *B. bacteriovorus* in chickens infected with *S. enterica*, and (2) their topical applications in cows suffering from *Moraxella bovis* infections (Atterbury et al., 2011; Boileau and Clinkenbeard, 2011). It is important to note that individual BALOs are tolerant, or even immune, to some toxic, antibiotic, or antiseptic agents due to the presence and activities of specific efflux pumps (Markelova, 2002). Even in human saliva, which acts as an antibacterial agent through protective and antimicrobial proteins (e.g., peroxidases, mucins, or lysozymes), some BALOs are able to retain their activities (Slowey et al., 1968; Van Nieuw Amerongen et al., 2004).

**FIGURE 5:**
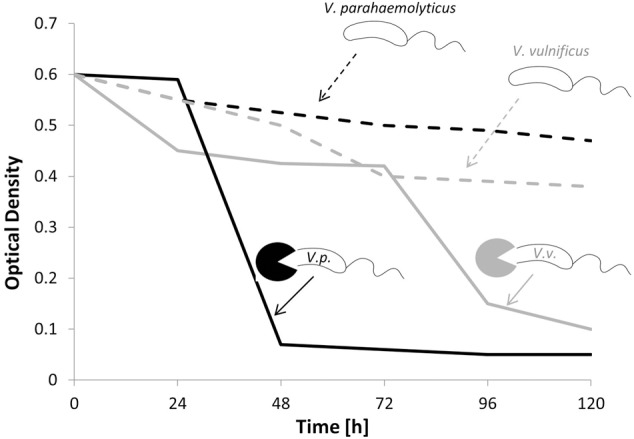
Concentrations of *Vibrio parahaemolyticus* and *Vibrio vulnificus* in the presence (solid black and gray line) and absence (dashed black and gray line) of bacterivorous *Bacteriovorax* (modified from Chen et al., 2011).

Studies addressing the application of bacterivorous bacteria in specific ecosystems are scarce, and little is known about the quantitative effects of bacterial predation on pathogens. Like other antagonistic processes, previous research was mainly restricted to the well-defined laboratory experiments rather than the field studies. In a microcosm experiment, the BALO *Bacteriovorax* was inoculated simultaneously with two pathogenic *Vibrio* species (*V. vulnificus* and *V. parahaemolyticus*) and the change in optical density (OD) was monitored over a period of 120 h (**Figure 5**). During this time, a constant decrease in OD was observed, indicating a reduction in the *Vibrio* strains. As depicted in **Figure 5**, *Bacteriovorax* revealed a higher preference toward *V. parahaemolyticus* (Chen et al., 2011). A successful application of BALOs in an aquaculture system was documented by Chu and Zhu (2010), where induced *Aeromonas hydrophila* infections in fishes were cured by the administration of *B. bacteriovorus*. Considering the very few environmental applications, a better and fundamental understanding of the role of BALOs in natural aquatic ecosystems is greatly desired.

## Potential Limitations

When entering the natural aquatic environment, pathogens become involved in food web interactions and competition, and by that become victims of the “microbial war” (Hibbing et al., 2010). In contrast, microorganisms, in particular, bacteria and viruses, exhibit an immense drive for developing adaptations to cope with unfavorable conditions or changing environments. Thereby, microorganisms (including pathogens) develop protections and resistance measures for circumventing one or more antagonistic processes, whether they are abiotic or biotic. Besides the fact that natural microbial communities in the aquatic environments respond antagonistically to human pathogens, and the fact that there are multiple lines of evidence from laboratory studies indicating that biocontrol is indeed possible, translating the potential effects of individual antagonistic interactions into the complex natural aquatic environment at this time seems difficult, at best. In most studies, antagonistic effects of microbes and phages on certain pathogens were evaluated either under well-defined laboratory conditions examining only isolated processes, or at only a descriptive level. Moreover, in most of these studies, fecal-indicator organisms, as well as model bacteria and viruses, were applied almost exclusively at concentrations exceeding the typical expected abundances of pathogens in the environment by orders of magnitude. As a consequence, various limitations require serious consideration. In the following, we briefly discuss the types of defense and resistance mechanisms that may be developed by pathogens against the antagonistic organisms and their agents. Moreover, additional practical shortcomings and risks associated with the active field applications are mentioned.

Prokaryotes may efficiently escape the pressures from various phages by developing infection resistance. Herein, bacteria and archaea have developed different strategies that reduce, or even inhibit, phage invasions, or at least minimize the associated effects. One prominent example is the recently discovered prokaryotic immune system, known as CRISPRs (clustered regularly interspaced short palindromic repeats). CRISPR is based on small RNAs (“spacers”) that restrict phage and plasmid infections (Barrangou et al., 2007; Labrie et al., 2010; Samson et al., 2013). There is also an increasing evidence that points toward bacterial quorum sensing (QS), a form of bacterial signaling that allows gene expression regulation to be involved in modulating the phage response (Høyland-Kroghsbo et al., 2013; Tan et al., 2015; Qin et al., 2016). Although data on the evolutionary rates for developing phage resistance and mechanisms for the development of new infection strategies by phages are rare, reports of rapid appearances of resistant host mutants within days to weeks deserves consideration (Padan and Shilo, 1973; Barnet et al., 1981; Tucker and Pollard, 2005).

Since viruses are incapable of active movement, they encounter their hosts by passive transport and diffusion. As a result, greater abundances of suitable hosts lead to higher encounter rates between viruses and their bacterial hosts (De Paepe et al., 2014). As such, certain host and phage densities are required for successful phage infections to occur, resulting in the death of the host population (Chibani-Chennoufi et al., 2004). Active applications of phage therapy against pathogens in the aquatic environment, therefore, involve the production of large amounts of active inoculum, as well as appropriate quantitative distribution to the target hosts. Regardless, it is unlikely that lytic phage activities will ever result in the complete elimination of their targeted hosts, as drastic reductions in the host density reduce the chances of phages successfully encountering new host cells. Besides, phages often have a narrow host range, making a prior identification of the causative bacterial agent necessary. Another concern is the potential toxic effects of components released from lysed pathogenic bacteria.

To successfully apply phage therapy for the control of pathogens in the environment, several strategies can be implemented to overcome certain limitations and to increase the efficacy. First, phage cocktails containing a mixture of several lytic phages may be used to broaden the susceptible host range. Second, a combination of several lytic phages collectively with antimicrobials may be favorable to prevent the rapid development of resistance. Third, specifically engineered bacteriophages are a promising option as well, providing benefits such as expressing EPS-degrading enzymes, expressing certain receptor-binding domains during their infection cycle, or delivering dominant genes that reverse the bacterial antibiotic resistance (Marzari et al., 1997; Lu and Collins, 2007; Edgar et al., 2012; Viertel et al., 2014).

As aforementioned, the effectiveness of protozoa as antagonistic agents against pathogens highly depends on their growth and grazing rates, their specialization for the prey, as well as the grazing pressure faced by the predators from higher organisms, such as copepods (Sigee et al., 1999 and references therein). It is also well-known that, facing grazing pressure, some bacteria escape from or compensate for predation by physiological and morphological adaptations (Hahn and Höfle, 2001; Justice et al., 2008). Recently, a correlation has been observed between the development of resistance against protozoan grazing and an increase in virulence (Adiba et al., 2010).

The resistance of microbes against antimicrobials (e.g., antibiotics) has been extensively studied and there is no doubt that bacteria may develop immunity against specific drugs after a period of exposure (Tenover, 2006). Moreover, a drug-specific immunity may be spread and shared with others through plasmid conjugation or horizontal gene transfer (Rosenblatt-Farrell, 2009). Nevertheless, the selective force leading to the resistance toward different antimicrobials is directly related to their absolute concentrations and times of exposure. In the environment, exposures may be transient and concentrations are rather low. Assuming that human pathogens, initially exposed to the environment, are non-growing and under physiological stress, they may be unable to develop resistance in the first instance upon exposure to antimicrobials. Alternatively, low concentrations caused by the dilution of extracellular excreted compounds may considerably limit the effectivity. Since most molecules act in a concentration-dependent manner, it is worth mentioning that antimicrobial substances may, at lower concentrations, also act as chemical signals in inter- and intracellular communication (Yim et al., 2007; Hibbing et al., 2010).

Application of these concepts requires knowledge of threshold concentrations of lytic bacteria and compounds, as well as an understanding of the spatial vicinity of the antagonist and the pathogen (e.g., Fallon and Brock, 1979). Notably, the processes leading to the inactivation and elimination of pathogens also may shape their genetic diversities, assuming that the more persistent pathogens are transmitted to the human host and later may re-enter the environment, becoming a second generation of pathogen. In face of the increasing number of multidrug-resistant bacteria, the environmental application of antimicrobial substances at high concentrations must be handled with caution.

While the resistance of microbes against BALOs remains unclear, experiments in chemostats have revealed the occurrence of bacteria that are transiently resistant to BALOs; however, in the absence of a predator, resistant bacteria were outcompeted quickly by susceptible bacteria, indicating that resistance is more of a plastic phenotypic response, rather than a mutational event (Varon, 1979; Shemesh and Jurkevitch, 2004). Although studied for decades now, our understanding of the entire biology of BALOs is still rather incomplete, which greatly limits its targeted application in the field. The mechanisms, by which the predatory bacteria identify a suitable prey, while beneficial bacteria are unaffected, remain cryptic. For an active application, their unspecific operating modes and their broad range of hosts must be considered (Dwidar et al., 2012).

Facing the potential repertoire and diversity of resistance mechanisms that microbes may develop, successful and sustainable applications of natural antagonists is challenging. Indeed, biological control may represent only a short-term measure for reducing the unwanted populations of microbes and viruses (Sigee et al., 1999). Long-term control strategies may need to involve steering abiotic factors, such as wastewater load or nutrient limitations (bottom–up control). Complementary effects of abiotic environmental factors can be detrimental or beneficial to pathogens, as well as to the biological agents (phages, lytic bacteria, or grazers), and can contribute to the complexity and unpredictability of antagonistic processes and their targeted applications; an aspect that, in particular, awaits consideration in future research. Integrative strategies based on physical, chemical, and biological processes are most promising.

Eventually, it is important to consider that most human pathogens entering aquatic habitats experience unfavorable, or even hostile, environmental conditions, preventing significant reproduction and posing physiological stress. This fact may reduce the likelihood that pathogens acquire resistance. Alternatively, global climate change scenarios, leading to warmer waters and increased nutrient loads, may trigger the survival and reproduction of human pathogens in aquatic environments, such that biocontrol by antagonistic interactions will gain a greater importance in the near future.

## Conclusion

Biotic antagonistic mechanisms interfere with the propagation and survival of pathogenic microorganisms in the aquatic environment. Since environment-based data are rare, a preliminary evaluation of contributions of the microbial and viral antagonists in inactivating and eliminating pathogens is possible presently on a qualitative scale only. To the best of our knowledge, lytic phages, predatory bacteria, as well as antimicrobial substances produced by autochthonous bacteria promise a broad range of applications, not only for the medical and food industries but also as a means of controlling and restoring the aquatic environment. Applying a combination of several mechanisms will increase the effectiveness of the methods used, and will broaden the range of susceptible pathogens being targeted. In face of the rapidly increasing number of multidrug-resistant microbes, further discoveries in the field of microbial antagonistic interactions are urgently needed.

## Author Contributions

CG, JF, and LD conceived the idea for the manuscript. JF and CG wrote the manuscript. LD substantially commented on and edited the manuscript.

## Conflict of Interest Statement

The authors declare that the research was conducted in the absence of any commercial or financial relationships that could be construed as a potential conflict of interest.
